# Supplementary material for: Association between oral microbiome and seven types of cancers in East Asian population: a two-sample Mendelian randomization analysis
Source: Front Mol Biosci. 2023 Nov 21;10:1327893. doi: 10.3389/fmolb.2023.1327893 (PMC10702768; doi:10.3389/fmolb.2023.1327893)
Supplement: Supplementary file 1 [file DataSheet1.ZIP › Supplementary Materials/MR plots for tongue/tongue═╝/Ovarian cancer/pheno.2607_to_ovarian cancer_leave_one_out.pdf]

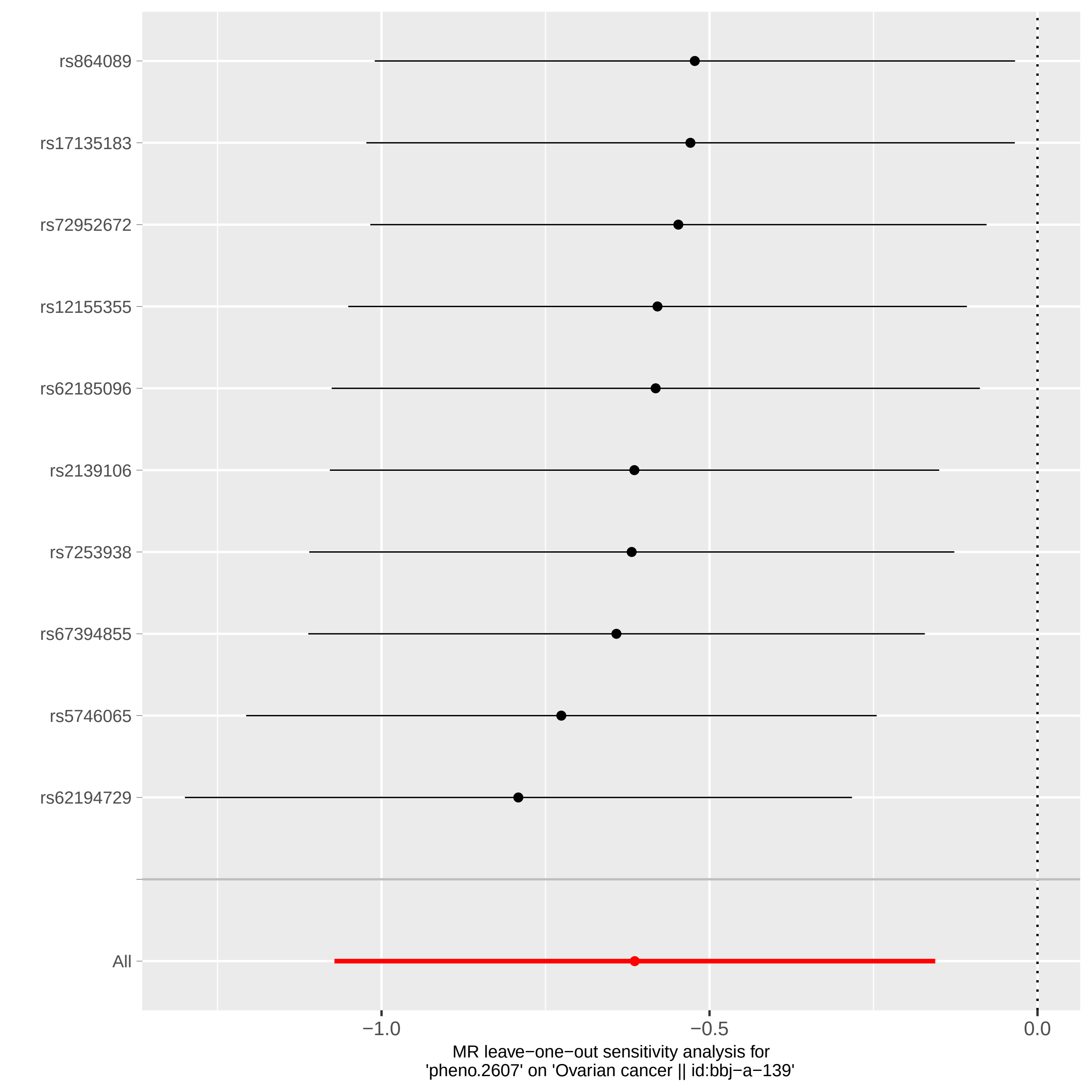
rs864089
rs17135183
rs72952672
rs12155355
rs62185096
rs2139106
rs7253938
rs67394855
rs5746065
rs62194729
All
−1.0
−0.5
0.0
MR leave−one−out sensitivity analysis for
'pheno.2607' on 'Ovarian cancer || id:bbj−a−139'
